# In depth characterisation of the proteome of MIS-C and post COVID-19 infection in children reveals inflammatory pathway activation and evidence of tissue damage

**DOI:** 10.1186/s12967-025-06826-3

**Published:** 2025-08-18

**Authors:** Cathal Roarty, Claire Tonry, Claire McGinn, Sharon Christie, Tom Waterfield, Chris Watson

**Affiliations:** 1 Wellcome-Wolfson Institute for Experimental Medicine, Queen’s University Belfast, Belfast, BT97BL UK; 2 Royal Belfast Hospital for Sick Children, Belfast, BT97BL UK

## Abstract

**Background:**

Multisystem inflammatory syndrome in children (MIS-C) is a rare but severe complication that arises between two and six weeks after initial SARS-CoV-2 infection. The mechanisms underlying why only a subset of children develop this hyperinflammatory response remain unclear.

**Methods:**

We performed an in-depth proteomic analysis of plasma samples from children before and after SARS-CoV-2 infection, including those who developed MIS-C. Proteomic profiling was conducted using high-throughput technologies, and findings were validated using publicly available datasets.

**Results:**

Healthy children showed minimal changes in the circulating proteome following SARS-CoV-2 infection, with no evidence of ongoing inflammation. In contrast, children with MIS-C exhibited significant activation of pro-inflammatory pathways and elevated circulating markers of myocardial and vascular injury.

**Conclusions:**

Our data suggest that SARS-CoV-2 infection alone does not cause sustained proteomic alterations in most children. However, MIS-C is associated with a distinct inflammatory and vascular injury signature. Several candidate diagnostic biomarkers for MIS-C were identified and validated in silico, offering promising avenues for future diagnostic and therapeutic strategies.

**Supplementary Information:**

The online version contains supplementary material available at 10.1186/s12967-025-06826-3.

## Graphical Abstract

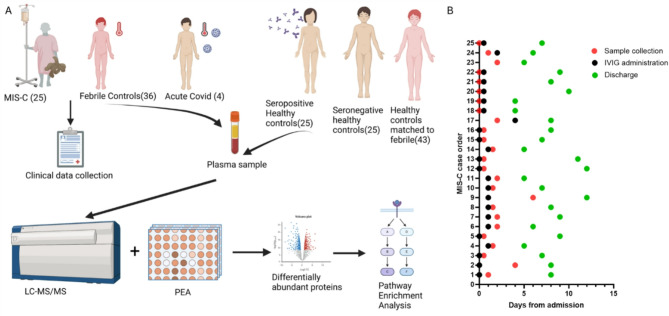

## Introduction

SARS-CoV-2 is a respiratory virus that can manifest as a mild or, in some instances, fatal disease. Most SARS-CoV-2 infections in children are mild, and do not require hospitalisation [1, 2]. A small number of children, however, progress to develop Multisystem Inflammatory Syndrome in Children (MIS-C) in the weeks following infection with SARS-CoV-2 [3–7].

Clinically, MIS-C shares many features of Kawasaki Disease (KD) and macrophage activation syndrome, with fever present in all cases, along with gastrointestinal symptoms, mucosal changes, and shock in many cases [1, 7–10]. Whilst the pathogenesis of MIS-C is poorly understood, it is thought that a cytokine storm and T cell hyperactivation play significant roles [11–17]. Expansion of a specific subset of T-cells expressing the receptor TRBV11-2 has been identified in a number of studies, although this is only present in a proportion of cases. The humoral response to SARS-CoV-2 in MIS-C is broadly comparable to the response in children and adults who have not progressed to a hyperinflammatory state, although some differences in the populations of SARS-CoV-2 specific B cells have been identified previously. Similar to other childhood inflammatory conditions, current therapies include immunomodulation with intravenous immunoglobulin (IVIG) and/or steroids [18–20]. The precise therapeutic mechanism of action for both IVIG and steroids in MIS-C is poorly understood.

Several studies have examined the proteome of children acutely unwell with MIS-C, with reports of elevations in circulating pro-inflammatory cytokines, increased platelet activation, and evidence of endothelial damage [14, 21–24]. However, very little is known about the convalescent proteome of children following SARS-CoV-2 infection, and by extension differences in the circulating proteome of these children and children who have progressed to MIS-C have not been established. As MIS-C is a post infection condition, a convalescent, healthy cohort would provide the best available representation of a healthy proteome in the period following infection with which to compare the aberrant response of MIS-C. There has yet to be a comparison made between the circulating proteome of children who have fully recovered following SARS-CoV-2 and the circulating proteome of children who developed MIS-C. Furthermore, there have not yet been any studies comparing the proteome of children with MIS-C and the proteome of children presenting with a febrile illness caused by another viral or bacterial pathogen.

Hence, the objective of this study was to describe the proteome of children under 16 years of age with MIS-C and to compare that to age matched healthy controls, febrile controls with a similar disease severity, and to children convalescing from acute SARS-CoV-2 infection.

Here we harnessed the ability of two proteomic technologies - data independent acquisition (DIA) LC-MS/MS and proximity extension assays (PEA) to achieve comprehensive coverage of the circulating proteome, in order to fully characterise the proteomic signature of children following SARS-CoV-2 infection, including the proteome of MIS-C. We have identified dysregulated biological pathways which are reflected in the clinical presentation of MIS-C and a distinct proteomic signature for MIS-C. We identify promising candidate diagnostic biomarkers for MIS-C and verify these in publicly available datasets.

## Results

### Patient cohort

A total of 158 samples were included for this study from pediatric patients aged between 3 and 191 months. Children were recruited from the Royal Belfast Hospital for Sick Children as part of the Covid Warriors study, details of which have been published previously [25]. The cohort includes 25 children with MIS-C, 4 children hospitalised with acute COVID-19, 36 hospitalised febrile controls, 25 SARS-CoV-2 seronegative healthy controls (14 of whom have a seropositive sample from a later blood draw), 25 SARS-CoV-2 seropositive healthy controls, and a further 43 healthy controls recruited as part of the Covid Warriors study, matched to febrile controls. The demographic characteristics of these cohorts are described in Table 1, and the clinical characteristics of the hospitalised cohorts are described in Table 2. Further details on the MIS-C and febrile control cohorts are available in the supplementary material. An illustration of the sample cohorts used is shown in the graphical abstract.

**Table 1:** Overall demographics of cohorts used in this study

	MIS-C (*n* = 25)	Febrile Controls (*n* = 36)	Acute COVID-19 (*n* = 4)	Seronegative Healthy controls (*n* = 25)	Seropositive Healthy controls (*n* = 25)	Healthy controls matched to febrile (*n* = 43)	*P* Value
**Sex**	13 M/12F	18 M/18F	1 M/3F	13 M/12F	12 M/13F	20 M/23F	
**Age**(median (IQR))	9.92(6.75–13.75)	4.96(1.7–9.83)	5.7(1.56–13.75)	9.25(5.635–13.59)	9.75(6.8–13.0)	4.74(3.62–9.98)	< 0.01
**PICU admission**(%)	11(44%)	18(50%)	2(50%)	NA	NA	NA	

**Table 2:** MIS-C, febrile controls and acute Covid patients’ clinical characteristics

Clinical characteristics				
Clinical characteristic			COVID-19, *N* = 4^1^	*p*-value^2^
	**MIS-C**, *N* = 25^*1*^	**Febrile Controls**, *N* = 36^*1*^		
**Pre-existing health condition**	6 (24%)	21 (60%)	2 (50%)	0.015
**Regular medication**	2 (8.0%)	7 (19%)	1 (25%)	0.31
**Maximum CRP (mg/L)**				< 0.001
Median (IQR)	221 (158, 283)	67 (29, 114)	NA	
**Steroid administered**	18 (72%)	10 (28%)	3 (75%)	< 0.001
**Antibiotic administered**	9 (41%)	31 (86%)	3 (75%)	< 0.001
**Inotrope administered**	10 (40%)	6 (17%)	1 (25%)	0.12
**Respiratory support**	6 (27%)	24 (67%)	3 (75%)	0.006
**Invasive ventilation**	3 (17%)	17 (47%)	1 (25%)	0.077
**Length of hospital stay(days)**				0.97
Median (IQR)	8.0 (6.0, 9.0)	7.0 (5.0, 14.8)	7.0 (7.0, 7.0)	
**Outcome**				0.15
Died	0 (0%)	2 (5.6%)	1 (25%)	
Survived	25 (100%)	34 (94%)	3 (75%)	

^*1*^ n (%)

^*2*^ Kruskal-Wallis rank sum test; Fisher’s exact test

### Structure of the proteomic datasets

**Fig. 1:**
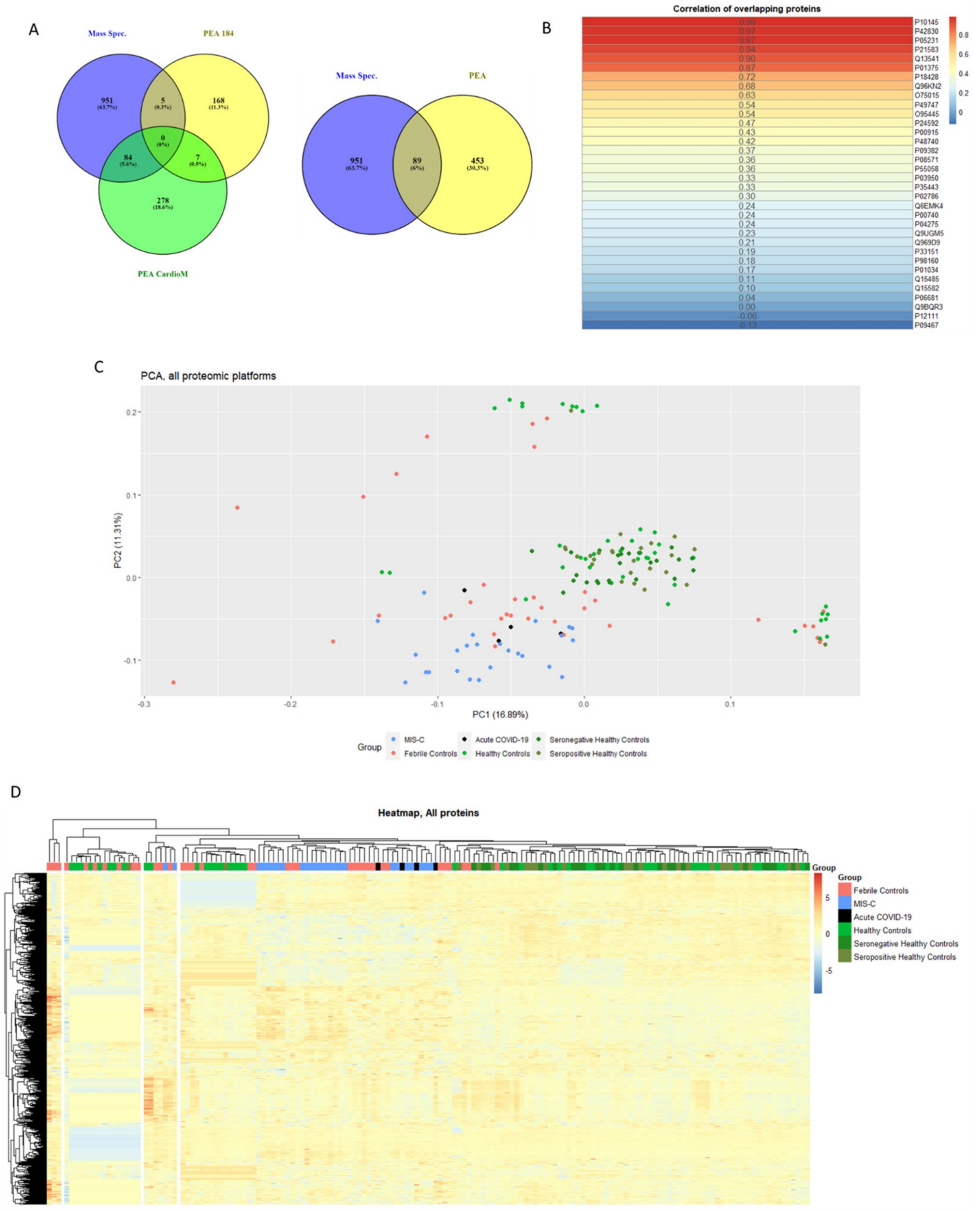
(**A**) Venn diagram of overlap of proteins measured by LC-MS/MS and the proximity extension assays (PEA) panels prior to missing value filtering. PEA 184 refers to the Olink Inflammation target 96 and Immune response target 96 panels, PEA 384 refers to the Olink Cardiometabolic explore 384 panel. (**B**) Heatmap of Pearson’s correlation coefficients of the 34 proteins measured by both LC-MS/MS and PEA after filtering for missing data.(**C**) Principal Component Analysis (PCA) of combined proteomic data. (**D**) Unsupervised hierarchical clustering based on all differentially abundant proteins

Two proteomics technologies were applied to maximise coverage of the plasma proteome. An unbiased diaPASEF mass spectrometry analysis was used to detect medium to low-abundant plasma proteins. This led to the identification of 1,040 proteins across all samples. After filtering for missing data, 291 proteins quantified in ≥ 70% of samples (*n* = 111) were retained, with 7.86% missing values. PEA-based analysis was performed by Olink, using three of their most disease relevant panels, for more sensitive detection of very low abundant proteins. This resulted in detection of 542 proteins. As shown in Fig. 1A there was minimal overlap in proteins identified from both platforms, thereby demonstrating the benefit ofcombining proteomic technologies. Eighty-nine proteins were initially identified by both LC-MS/MS and PEA; however, after filtering the LC-MS/MS dataset for proteins with < 30% missing values, 34 overlapping proteins remained. Of these, 20 proteins (58.8%) showed a Pearson correlation coefficient ≥ 0.3, suggesting moderate concordance between platforms (Fig. 1B). In total 1,491 plasma proteins were identified. We employed principal component analysis to provide an unbiased analysis of the structure of the proteome in our combined dataset (Fig. 1C). The MIS-C and febrile control groups separated from the proteome of the healthy control groups, with some overlap between the MIS-C, Acute COVID-19 and febrile control groups. Among the top contributing proteins to component one, which separated the hospitalised groups from the healthy controls were Chemokine CC motif ligand 7 (CCL7), interleukin 6 (IL-6) and tissue inhibitor of metallopreinase 1 (TIMP-1).

### Proteome profiling following SARS-CoV-2 infection in healthy children

**Fig. 2:**
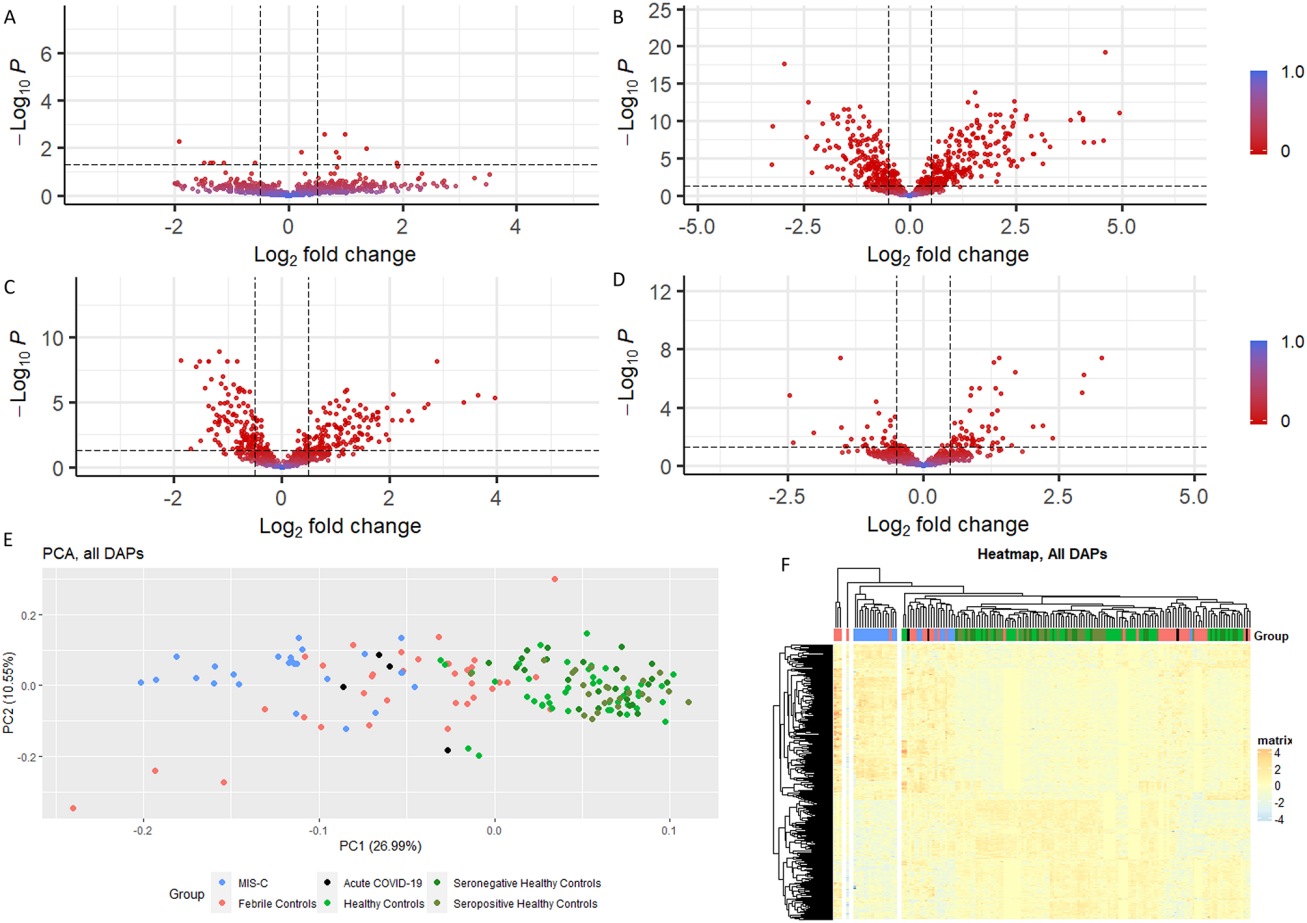
(**A**) Volcano plot showing Differentially Abundant Proteins(DAPs) between Acute COVID-19 (*n* = 4) and matched seronegative healthy controls (*n* = 25) (**B**) Volcano plot showing DAPs between MIS-C(*n* = 25) and seropositive healthy controls (*n* = 25) (**C**) Volcano plot showing DAPs between febrile controls (*n* = 36) and matched healthy controls (*n* = 43). (**D**) Volcano plot showing DAPs between MIS-C (*n* = 25) and febrile controls (*n* = 36). (**E**) Principal Component Analysis of DAPs only (*n* = 463). (**F**) Unsupervised hierarchical clustering based on all DAPs (*n* = 463)

We compared the proteome of children hospitalised with acute COVID-19 with a matched seronegative healthy control cohort, identifying a number of differentially expressed proteins, which were related to TNF pathway activation, and L1 cellular adhesion molecule interactions (Fig. 2A) (Supplementary Fig. 1).

To investigate changes in the circulating proteome following SARS-CoV-2 infection amongst healthy children, we analysed samples collected pre and post SARS-CoV-2 infection from unvaccinated children, with median time between initial seronegative sample and the following seropositive sample being 181 days (IQR 134 to 201). There were no significantly differentially abundant proteins (DAPs) between these groups (Supplementary Fig. 2).

### Proteomic changes associated with MIS-C

Three hundred and eighty nine DAPs were found between MIS-C and seropositive healthy controls, 172 downregulated in MIS-C and 217 upregulated (Fig. 2B). There were 140 downregulated and 149 upregulated significant DAPs between the febrile control group and the healthy control group (Fig. 2C). Comparison of the MIS-C group and the febrile controls identified 93 differentially abundant proteins,60 upregulated in MIS-C in comparison to the febrile control group and 33 downregulated in MIS-C (Fig. 2D).

To better visualise the differences between the groups we performed a PCA using only the 463 DAPs identified in at least one of the comparisons described above (Fig. 2E). Greater separation between groups was achieved with this subset of proteins, and hierarchical clustering revealed a single cluster of MIS-C cases (Fig. 2F). This indicates that MIS-C has a unique proteomic profile in comparison to healthy and febrile control cases.

### Biological interpretation of MIS-C proteome

**Fig. 3:**
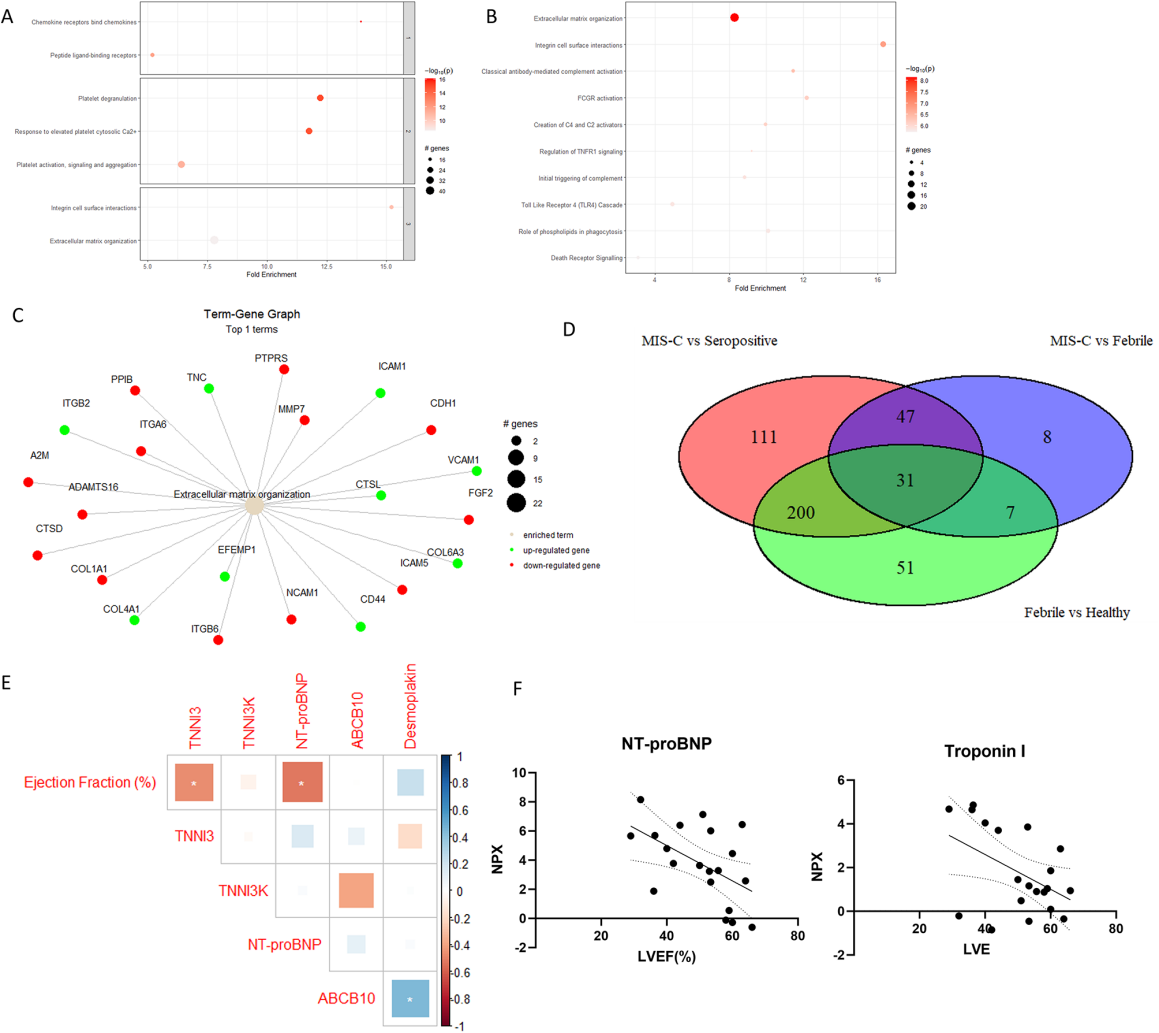
(**A**) Enriched pathways in MIS-C in comparison to seropositive healthy controls, top 3 Reactome pathway clusters shown. (**B**) Enriched pathways pathways in MIS-C in comparison to febrile controls. (**C**) Term-Gene graph displaying DAP’s between MIS-C and febrile controls associated with the reactome pathway Extracellular matrix reorganisation. (**D**) Venn diagram showing proteins which are significantly differentially abundant between MIS-C and seropositive healthy controls only, between febrile controls and healthy controls only, and DAPs between both MIS-C and seropositive healthy controls, and febrile controls and healthy controls. (**E**) Correlation of markers of myocardial damage and initial left ventricular ejection fraction (%) of MIS-C cases(*n* = 20), Pearson’s correlation shown. (**F**) Scatter plot of abundance of circulating NT-proBNP, Troponin I and initial left ventricular ejection fraction (LVEF) (%) of MIS-C cases(*n* = 20)

An enrichment in pathways involving chemokine signalling, platelet degranulation and integrin signalling pathway activation were identified in MIS-C in comparison to seropositive healthy controls (Fig. 3A). No DAPs were identified between MIS-C cases who received IVIG prior to sample collection (*n* = 9) and those who had not (*n* = 16) (Supplementary Fig. 4). Pathway analysis revealed enrichment of pathways in MIS-C that were not enriched in febrile controls, a number of which were also found to be enriched in MIS-C in the comparison to seropositive healthy controls (Fig. 3B). Evidence for endothelial damage, which has been previously reported as a feature of MIS-C, was present in our cohort, with increased Vascular endothelial growth factor A (VEGFA), Vascular Cellular Adhesion Molecule 1 (VCAM1) and E-selectin levels in MIS-C cases in contrast with seropositive healthy controls [23] (Supplementary Fig. 5A). Extracellular matrix organization was the most significantly enriched pathway in MIS-C in comparison to febrile controls, with increased levels of Tenascin C in MIS-C, while fibroblast growth factor 21 (FGF21), which attenuates cardiac remodelling is decreased (Fig. 3C). By comparing the significantly differently abundant proteins between the febrile controls and their matched healthy controls, and the differentially abundant proteins between the MIS-C cohort and the matched seropositive healthy controls, 111 proteins that are uniquely associated with MIS-C and not significantly deregulated in other febrile disease cases were identified (Fig. 3D). These proteins included Leukocyte immunoglobulin-like receptor subfamily B member 2 (LILRB2), prostaglandin D2 synthase (PTGDS), T cell immunoglobulin and mucin domain containing 4 (TIMD4), Fc gamma receptor IIIb (FCGR3B) and Tumor Necrosis Factor Alpha (TNF- α) (Supplementary Fig. 5D). Reflecting the cardiac involvement that has been observed in MIS-C cases, levels of Troponin I, Troponin I3 kinase (TNNI3K), Extracellular newly identified receptor for advanced glycation end products binding protein (EN-RAGE) and N-terminal pro-brain natriuretic peptide (NT-proBNP) were found to be increased in this MIS-C cohort, while ATP binding cassette subfamily B member 10 (ABCB10) is decreased in comparison to seropositive controls (Fig. 3E). A significant negative correlation between the initial left ventricular ejection fraction (LVEF) of MIS-C cases and the levels of Troponin I and NT-proBNP was found, indicating a decrease in cardiac output due to an acute insult to the myocardium in MIS-C (Fig. 3F).

### Candidate diagnostic proteins of MIS-C

**Fig. 4:**
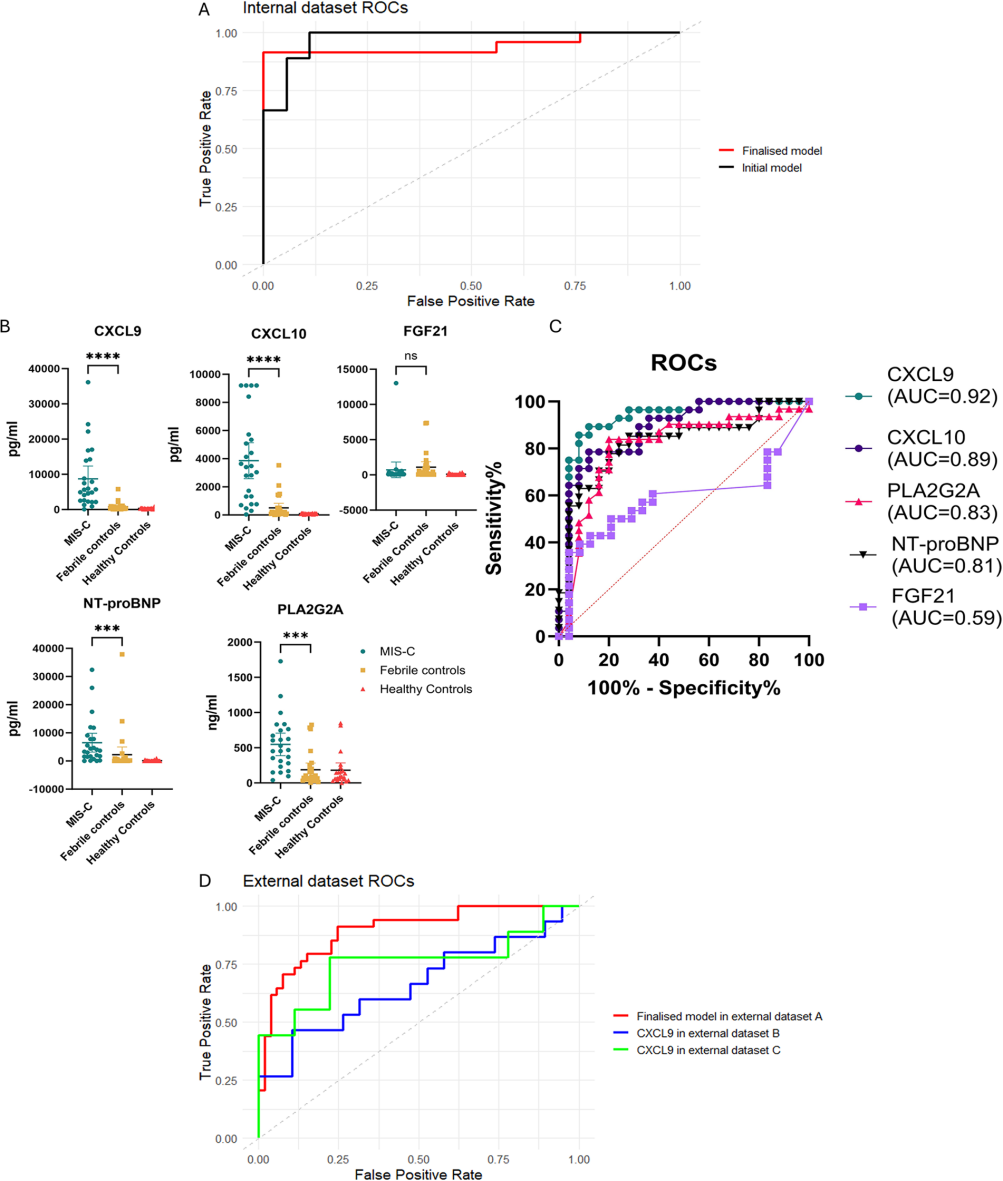
(**A**) Receiver operating characteristic (ROC) curves of initial LASSO model performance in discriminating MIS-C in the test set using proteomics data for the 5 selected candidate markers (CXCL9, CXCL10, PLA2G2A, FGF21, and NT-proBNP), and the finalised models performance in discriminating MIS-C from febrile controls in the ELISA generated data. (**B**) Plasma concentration of candidate markers in MIS-C (*n* = 25), febrile controls (*n* = 27) and healthy controls (*n* = 21) compared by Kruskal-Wallis one way ANOVA with multiple comparisons. *P < 0.05, **P < 0.01, ***P < 0.001, ****P < 0.0001. Median values and interquartile range are shown. (**C**) ROC curves of each individual candidate diagnostic marker quantified by ELISA. (**D**) ROC curves of finalised model performance in external dataset A, and performance of one of the 3 proteins in the model, CXCL9, in external dataset B and external dataset C [21–23]

Top DAPs identified in this study were evaluated as candidate diagnostic markers to support the differentiation between MIS-C and febrile controls in our cohort. The nine proteins with an absolute log_2_ fold change > 2 and FDR-adjusted *p* < 0.05 in this comparison showed consistent directionality of fold changes when evaluated across external datasets (Supplementary Fig. 7) [21–23]. Using the least absolute shrinkage and selection (LASSO) method we identified 4 proteins which discriminated between MIS-C and febrile controls: CXCL9, CXCL10, PLA2G2A, and FGF21. An additional protein was included, NT-proBNP, as it was differentially abundant in comparison to the febrile control group and is available as a widely used clinical assay. LASSO regression modelling performance was assessed in a test data set, achieving an AUC of 0.976 (Fig. 4A). To verify the difference in abundance of these five candidate protein biomarkers between MIS-C and the febrile control group, ELISA’s were used to quantify the plasma concentrations of the proteins in these cohorts.

When measured by ELISA, the median plasma concentrations of CXCL9, CXCL10, PLA2G2A and NT-proBNP were significantly higher in MIS-C than in febrile controls (*p* < 0.0001, *p* < 0.0001, *p* < 0.0001, *p* = 0.004) (Fig. 4B). Median plasma concentration of FGF21 was lower in MIS-C than in febrile controls, although not significantly (*p* > 0.999). Each markers individual ability as a classifier was assessed using receiver operating characteristic (ROC) curves, with the highest area under the curve (AUC) of 0.92 (95%CI 0.84-1.00) for CXCL9 (Fig. 4C). Statistically optimal cut-offs for each protein were determined by Youden’s index (Table 3). A further, finalised LASSO model was trained using this ELISA data, which selected three proteins, CXCL9, PLA2G2A and NT-proBNP, achieving an AUC of 0.945 (Fig. 4A).

**Table 3:** Optimal cutoffs for each protein marker for prediction of MIS-C compared to febrile controls, determined individually by youden’s index

Predictor	Optimal cutoff	Sensitivity	Specificity	AUC
CXCL10 (pg/ml)	>=1664.36	0.68	0.93	0.89
CXCL9 (pg/ml)	>=1448.99	0.88	0.89	0.92
FGF21 (pg/ml)	<=795.68	0.96	0.33	0.59
NT-proBNP (pg/ml)	>=2795.57	0.6	0.93	0.82
PLA2G2A (ng/ml)	>=235.77	0.76	0.8	0.83

We assessed the performance of this finalised model using an external publicly available proteomic dataset (external dataset A) which had 34 MIS-C cases and 43 cases of either severe or mild COVID-19 [23]. Our finalised model correctly identified 31 of the 34 (91.1%) as MIS-C, with an AUC of 0.896 (Fig. 4D). Two additional proteomic datasets (external dataset B and C) which had only data for CXCL9 available were evaluated, showing a relatively poorer performance in correctly classifying MIS-C although with differing comparison groups (Fig. 4D [21, 22].

## Discussion

This is the first study to compare the proteome of children with MIS-C to the proteomes of healthy children following resolved SARS-CoV-2 infection and febrile controls of a similar age and disease severity. In addition, this is the first utilisation of a combination of DIA LC-MS/MS and PEA thus providing the most comprehensive profile of the circulating proteome of children post SARS-CoV-2 infection that has been reported to date.

In our cohort of children hospitalised with COVID-19 we identified eight differentially abundant proteins in comparison with healthy seronegative children. Although a small cohort, proteins identified as dysregulated in other paediatric COVID-19 cohorts, such as the complement proteins C2, were also differentially abundant in this cohort [23, 26].

There were no significantly differentially abundant proteins between the seronegative and seropositive healthy children. This suggests that there is no permanent change in the circulating proteome in children following a mild SARS-CoV-2 infection, and that the sub clinical inflammatory processes which are proposed to cause long COVID are not present in this cohort [27–29]. There were no significant differences between the seronegative and seropositive children in the eight proteins which were significantly different in the acute COVID-19 cohort, which further reinforces that in healthy children following SARS-CoV-2 infection there is not a similar dysregulation of circulating proteins as to that in acute COVID-19.

The proteomic profile of MIS-C reveals a markedly different landscape compared to children infected with SARS-CoV-2 who remained asymptomatic. Our findings support previous reports identifying an exaggerated pro-inflammatory response in MIS-C, with elevated levels of mediators such as IL-6, TNF-α, CXCL9, and S100A9 [21–24, 30–33]. These observations are consistent with the hypothesis that MIS-C represents an overwhelming inflammatory state, although the precise pathogenesis remains to be elucidated.

We also identified significantly increased levels of cardiac-associated proteins, including NT-proBNP, troponin, and TNNI3K, indicative of myocardial involvement and potential cardiogenic shock [8, 9, 34–36]. The abundance of NT-proBNP and troponin correlated with reduced initial LVEF in MIS-C cases, supporting the hypothesis of myocardial stress or injury. While IVIG has been linked to fluid overload and elevated NT-proBNP in some cases, we observed no significant change in NT-proBNP levels before and after treatment in our cohort [37–39]. These findings, along with elevated markers of cardiomyocyte damage, suggest cardiac injury in MIS-C is likely secondary to systemic inflammation rather than direct viral myocarditis [34, 40].

Increased levels of CXCL9, CXCL10, and CXCL11 further support IFN-γ pathway activation in MIS-C, aligning with prior studies [23, 25]. Additionally, elevated markers of endothelial activation and platelet activation are consistent with the vasculitic features observed clinically [9, 41].

Although febrile controls shared some inflammatory signatures with MIS-C, the degree of activation was generally greater in MIS-C. Enrichment of extracellular matrix (ECM) organisation and remodelling pathways in MIS-C suggests acute tissue injury—particularly to the myocardium—and a downstream fibrotic or reparative response [42]. This may be linked to thrombotic microangiopathy, a proposed mechanism of endothelial injury in MIS-C [23, 43]. Reports to date show an improvement in cardiac function following recovery from MIS-C, with an associated decrease in some commonly measured circulating markers of myocardial insult [44, 45]. Complement pathway activation was also more pronounced in MIS-C and may reflect an autoimmune component, as supported by the detection of circulating autoantibodies in other cohorts [21, 22, 46].

Together, these findings suggest that MIS-C is characterised by a hyperinflammatory state with associated cardiac and vascular injury and extracellular matrix remodelling. Further investigation is warranted to define the triggers and sequence of these events, with the aim of building a mechanistic model of MIS-C pathogenesis.

The most differentially abundant proteins between MIS-C and febrile controls in our data were validated in silico with publicly available proteomic datasets [21–23]. Despite differences in disease control groups, analytical platforms, and geographic origins of these external datasets, the direction of fold change between MIS-C cohorts and healthy control cohorts for the majority of proteins was concordant with our findings, with only one dataset showing discordance. This cross-cohort consistency supports the robustness and generalisability of our results.

Candidate biomarkers were selected through penalized regression modelling and verified by ELISA in our cohort. Among these, CXCL9 consistently emerged as the top-performing marker, achieving the highest AUC in distinguishing MIS-C from febrile controls and receiving the strongest weighting in the final LASSO model. This reinforces previous reports of elevated CXCL9 in MIS-C cases in comparison with other pediatric febrile illnesses [23, 24, 33]. In our cohort, CXCL9 achieved the greatest AUC in discriminating MIS-C from the febrile control cohort and had the largest weighting in the finalised LASSO model. This finding is in agreement with a number of studies which suggest CXCL9 is a promising candidate diagnostic biomarker of MIS-C [47, 48]. In external dataset B dataset which had a Kawasaki disease cohort, CXCL9 was elevated in MIS-C compared with the Kawasaki cohort, consistent with the findings of increased abundance of CXCL9 in MIS-C cohorts compared with disease controls in our study and in the other datasets [21]. However, in external dataset B CXCL9’s discriminatory performance was modest (AUC 0.663) suggesting that this marker alone is not sufficient to distinguish these conditions reliably. Nevertheless, given that both conditions are managed with IVIG and corticosteroids, the more pressing diagnostic challenge lies in differentiating MIS-C from bacterial infections, particularly in children presenting with fever and abdominal symptoms [49].

PLA2G2A, a marker which has previously been suggested as a potential candidate biomarker for MIS-C, was also verified as elevated in our cohort [23, 50].

FGF21 was selected in the initial LASSO model; however, when measured by ELISA, its abundance did not differ significantly between MIS-C cases and febrile controls. As a result, it was not retained in the final LASSO model and is unlikely to represent a useful diagnostic marker for MIS-C.

Importantly, NT-proBNP, a clinically available marker, performed relatively well in discriminating MIS-C from febrile controls in our cohort. It has previously been suggested as a feature which discriminates MIS-C from other clinically similar conditions [51].

The performance of the finalised LASSO model in external dataset A, with an AUC of 0.896, is encouraging and provides preliminary support for the potential external validity of the candidate diagnostic proteins identified in this study [23].

There are a number of limitations to this study. A small number of MIS-C cases were included due to the rarity of the disease. Further independent validation of the candidate diagnostic proteins is required, with larger MIS-C and febrile cohorts. It is, however, the first study to profile the proteome of healthy children following SARS-CoV-2 infection, and to compare this with the proteome of MIS-C. It is also the first study to combine two orthogonal proteomic techniques, providing a wider coverage of the entire circulating proteome to characterise the proteomic changes incurred through SARS-CoV-2 infection and the unique proteome of MIS-C. The development of a diagnostic test for MIS-C must be contextualised within its rarity as a condition. A single standalone test is unlikely to be sufficient for diagnosis; instead, any potential biomarker should be integrated with established clinical diagnostic criteria [52, 53]. Evaluating the combined diagnostic performance of such biomarkers alongside clinical features would be the most appropriate approach.

Further validation of candidate biomarkers in larger, independent MIS-C and febrile illness cohorts is essential. Future work should also explore the diagnostic utility of these biomarkers in real-world clinical settings, particularly in distinguishing MIS-C from bacterial infections or other inflammatory syndromes at presentation, with a longitudinal component to establish whether these markers can guide treatment choices.

## Methods

### Recruitment

MIS-C and febrile controls were recruited from the Royal Belfast Hospital for Sick Children. Healthy controls were recruited as part of the Covid Warriors seroprevalence study and selected by matching on age and sex with the MIS-C cases [54]. An additional healthy control cohort was selected from the Covid Warriors study by age and sex matching with the febrile controls (Healthy controls matched for febrile).

### Case definitions

The Centers for Disease and Control criteria was used to define MIS-C. Febrile controls were children admitted to hospital with a history of fever greater than 38^o^C, with an identified microorganism.

### Clinical variables and data sources

Participants were screened for eligibility by clinical staff at the time of admission, and study data were recorded using an electronic CRF (supplementary material). All participants in the study received clinical care as per local guidance without delay. Study data included demographic details, clinical features, laboratory results, treatments received, final diagnosis, levels of care.

### Sample collection

Blood samples were collected by experienced clinical staff, contemporaneous with a routine clinical blood draw. Samples were collected in EDTA vacutainers and centrifuged within an hour of sample collection. Samples were spun at 1500xg and the plasma aliquoted into cryovials and stored at -80^o^C until required for analysis.

### Sample preparation for LC-MS/MS

Plasma samples processed and analysed by mass spectrometry as previously described [55, 56]. In brief, all samples were prepared in a 96 well-plate format which allows for medium throughput preparation of large sample numbers while minimizing technical variation. Thermofisher high select resin (product number A36372) was used in a 96 deep well filter plate format used for depletion of top 14 most abundant proteins. Crude plasma was aliquoted in a randomized order into wells containing resin, incubated while on an orbital shaker for 20 min at room temperature and then filter plates were placed on 96 well collection plates, and spun at 100xg for 5 min. The depleted plasma samples were vacuum concentrated and resuspended in 0.05 M ammonium bicarbonate, protein was acetone precipitated and resuspended in 8 M Urea, 0.1 M Tris-HCL pH 8.0. A sample pool of depleted samples was made. Sample protein concentration was measured using BCA (Pierce). 50ug protein equivalent of sample volume was aliquoted to a new 96 well plate and 5ul 100mM Dithiothreitol (DTT) added and incubated at 27 ^o^C for 1 h. 5ul 140mM iodoacetamide was added and incubated at room temperature for 30 min in the dark. 2ul 100mM DTT and 108ul 0.05 M ABC was added to dilute urea. 5ul Trypsin, (0.2ug/ul) was added and incubated for 16 h on thermomixer at 37 ^o^C. To quench the digest, 2ul of 100% Trifluoroacetic acid was added to a 1% final concentration. Samples were desalted through C18 resin [57]. A pool of digested samples was used for fractionation.

To prevent batch effects all samples were processed at the same time and measured on one LC-MS/MS run. All samples were analysed in a single experimental run on a timsTof Pro mass spectrometer (Bruker Daltonics) connected to a Evosep One liquid chromatography system (EvoSep BioSystems). A reversed-phase C18 Endurance column using preset 30 SPD method was used for peptide separation. Data dependent acquisition mode (DDA) was used for analysis of high pH-reversed phase fractionated sample pools to generate data for spectral library. Data independent acquisition mode (DIA) was used to analyse individual samples. Trapped ion Mobility spectrometry (TIMS) mode was used for data acquisition. Parallel accumulation serial fragmentation (PASEF) was used for selection of trapped ions for ms/ms. At regular intervals throughout the run pooled sample digests were analysed as a quality control. DIA-NN (Version 1.8.1) software was used to process raw data files for spectral library building, protein identification and quantification [58].

### Proximity extension assay

Plasma samples from MIS-C, febrile and healthy controls were randomized in a 96 well format. The Olink^®^ Target 96 Immune Response, Olink^®^ Target 96 Inflammation and Olink^®^ Cardiometabolic Explore proximity extension assay panels were used to measure 537 unique proteins [59] Each panel consists of a plate with each well containing 96 pairs of oligonucleotide tagged analyte specific antibodies. Four internal controls and six sample controls were run to allow for quality control of inter plate variation. The CT values from the qPCR reaction are reported as Normalised Protein Expression (NPX) values, which is an arbitrary unit on log2 scale.

### Measurement of CXCL9, CXCL10, FGF21

Plasma samples were quantified using the microfluidic ELLA (Protein Simple, CA, USA), with 4 analytes measured for each sample. Samples were diluted 1:10 with provided sample diluent and 50ul diluted sample aliquoted into well. A single well is used for each individual sample, with the microfluidic channels allowing triplicate measurement of each analyte from a single well.

### Measurement of NT-proBNP

A commercially available ELISA for NT-proBNP (Biomedica, Medizinprodukte GmbH) was used following manufacturer’s instruction. Plasma samples were diluted 1:10 with provided diluent, and ran in duplicate. Absorbance was measured at 450 nm using a Biomega colorimetric microplate reader and standard curve calculated.

### Measurement of phospholipase A2

Commercially available ELISA from ThermoFisher was used following manufacturer’s instruction. Plasma samples were diluted 1:3 with provided sample diluent and ran in duplicate, absorbance measured at 450 nm and standard curve calculated.

### Statistical analysis

Descriptive statistics for cohorts demographics show median values and interquartile range (IQR) for continuous data. Categorical data is expressed as proportions.

In comparisons between two groups or subgroups continuous data was compared by a Wilcoxon rank sum test. A chi-squared test for proportions of categorical data was used if there were more than 5 observations for each outcome, if not Fisher’s exact test was used.

LC-MS/MS protein intensities were log2-transformed, robustly filtered for missingness (> 70% non-missing values across all samples) and missing values were imputed based on a missing not at random approach, (median abundance of each protein minus 2 standard deviations) [60]. To account for inter-sample variation in total protein abundance, LC-MS/MS data, a robust median normalisation approach was applied to the filtered LC-MS/MS DIA data [61]. For each sample, the median protein intensity was subtracted from all protein values in that sample.

An unpaired t-test of the log normalised peak areas or normalised protein expression values was used for comparison between groups, with adjustment for multiple tests using the false discovery rate. A protein was considered significantly different with a FDR < 0.05 and a log2 fold change >+/- 0.5.

Hierarchical clustering was performed using pheatmap package, principal component analysis using factoextra and factomine R packages, volcano plots using Enhanced Volcano.

A least absolute shrinkage operator (LASSO) penalised regression model was used for variable selection. Proteins which had an FDR < 0.05 and a log2 fold change >+/- 2 between MIS-C and febrile controls were taken forward to LASSO. The MIS-C and febrile data was split 67% -33% into a training and test data set. Cross validation was used to choose the optimal lambda value, while alpha = 1. The model was trained in the training set. The coefficients generated by the trained model were used to select the variables to be included in a multivariable binary logistic regression model to classify samples in the test data set. Performance of the trained LASSO model, and the binary logistic regression model in the training and test sets were assessed using ROC’s. Optimal cut-off values were determined for each protein using Youden’s index.

### In silico validation

A set of proteins most differentially abundant between MIS-C and febrile control cohorts were identified as those with a fold change FDR < 0.05 and a log2 fold change >+/- 2.

PubMed was searched using terms “MIS-C”, “Proteomics”, “Pediatric Covid”, “LC-MS/MS”, and publicly available proteomic datasets accessed. Datasets were searched for most abundant MIS-C vs. febrile control proteins using uniprot and protein name. The identified differentially abundant proteins were compared with available comparison groups with an FDR corrected t-test, and a log2 fold change was calculated.

### Pathway enrichment analysis

Proteins from the combined dataset were inputted into PathfindR along with their respective log2fold change and unadjusted *p* value [62]. The Reactome pathway database was used for enrichment analysis(https://www.genome.jp/kegg/pathway.html). Using an active subnetwork enrichment analysis approach which accounts for protein-protein interactions, enriched pathways are identified from the protein list and outputted in table format. Pathways that had a *p*-value of < = 0.05 were considered significantly enriched.

## Electronic supplementary material

Below is the link to the electronic supplementary material.

Supplementary Material 1

Supplementary Material 2

Supplementary Material 3

Supplementary Material 4

Supplementary Material 5

Supplementary Material 6

Supplementary Material 7

Supplementary Material 8

Supplementary Material 9

Supplementary Material 10

Supplementary Material 11

Supplementary Material 12

Supplementary Material 13

Supplementary Material 14

## Data Availability

LC-MS/MS data is available on PRIDE (PXD051939). Data generated by PEA are available at 10.17632/m6zhcgrkmv.1. Anonymised clinical data is available in supplementary data.
