# Supplementary material for: In depth characterisation of the proteome of MIS-C and post COVID-19 infection in children reveals inflammatory pathway activation and evidence of tissue damage
Source: J Transl Med. 2025 Aug 18;23:929. doi: 10.1186/s12967-025-06826-3 (PMC12363030; doi:10.1186/s12967-025-06826-3)
Supplement: Supplementary file 11 — Supplementary Material 11 [file 12967_2025_6826_MOESM11_ESM.docx]

# Supplementary material

## Febrile controls clinical diagnoses

| ID | Diagnosis | Bacterial/Viral |
| --- | --- | --- |
| H003 | Aspiration pneumonia | Bacterial |
| H004 | VAP Multi organism pneumonia and bacteraemia | Bacterial |
| H005 | Appendicitis simple | Bacterial |
| H006 | Appendicitis complicated | Bacterial |
| H007 | Pneumonia - can't say viral or bacterial | Bacterial |
| H009 | UTI + bacteraemia | Bacterial |
| H012 | Appendicitis | Bacterial |
| H60 | Bacteraemia | Bacterial |
| H013 | Appendicitis complicated | Bacterial |
| H014 | Appendicitis - complicated | Bacterial |
| H015 | UTI | Bacterial |
| H016 | Appendicitis simple | Bacterial |
| H017 | ARDS following bacterial pneumonia | Bacterial |
| H018 | Bacterial pneumonia | Bacterial |
| H34 | Viral LRTI | Viral |
| H29 | RSV Bronchiolitis | Viral |
| H30 | RSV Bronchiolitis | Viral |
| H31 | Rhino virus LRTI + pneumothorax | Viral |
| H33 | Viral LTRI -RSV | Viral |
| H35 | Appendicitis complicated | Bacterial |
| H36 | RSV | Viral |
| H37 | Viral pneumonia | Viral |
| H38 | Viral pneumonia | Viral |
| H40 | RSV pneumonia | Viral |
| H42 | Viral gastroenteritis | Viral |
| H50 | Viral pneumonia | Viral |
| H51 | Bacterial pneumonia | Bacterial |
| H63 | Viral pneumonia | Viral |
| H66 | Bacterial pneumonia | Bacterial |
| H67 | Bacterial LRTI - sepsis | Bacterial |
| H68 | Viral LRTI | Viral |
| H56 | Rheumatic fever | Inflammatory |
| H57 | Rheumatic fever | Inflammatory |
| H64 | Croup | Viral |
| H65 | Quincy | Bacterial |
| H28 | Appendicitis | Bacterial |

## Proximity extension assay

There were 92 proteins quantified by the Inflammation assay and 92 proteins quantified by the Immune response assay. The Cardiometabolic assay quantified 369 proteins. Across all assays, 154 proteins were quantified in the plasma samples. There was an overlap between the assays, with 4 of the same proteins measured by both the “Inflammation” and “Immune Response” panels and 6 proteins measured by both the immune response and Cardiometabolic assays. Of the 4 proteins measured by both the inflammation and Immune response panels, the normalised protein expression values (NPX) were all significantly correlated, with a median Pearson’s r value of 0.96 (Figure 1). Similarly, of the 7 proteins which were analysed on either of the Inflammation or immune response panels and the cardiometabolic panel, 6 were significantly correlated with a median Pearson’s r value of 0.91 (Figure 2).


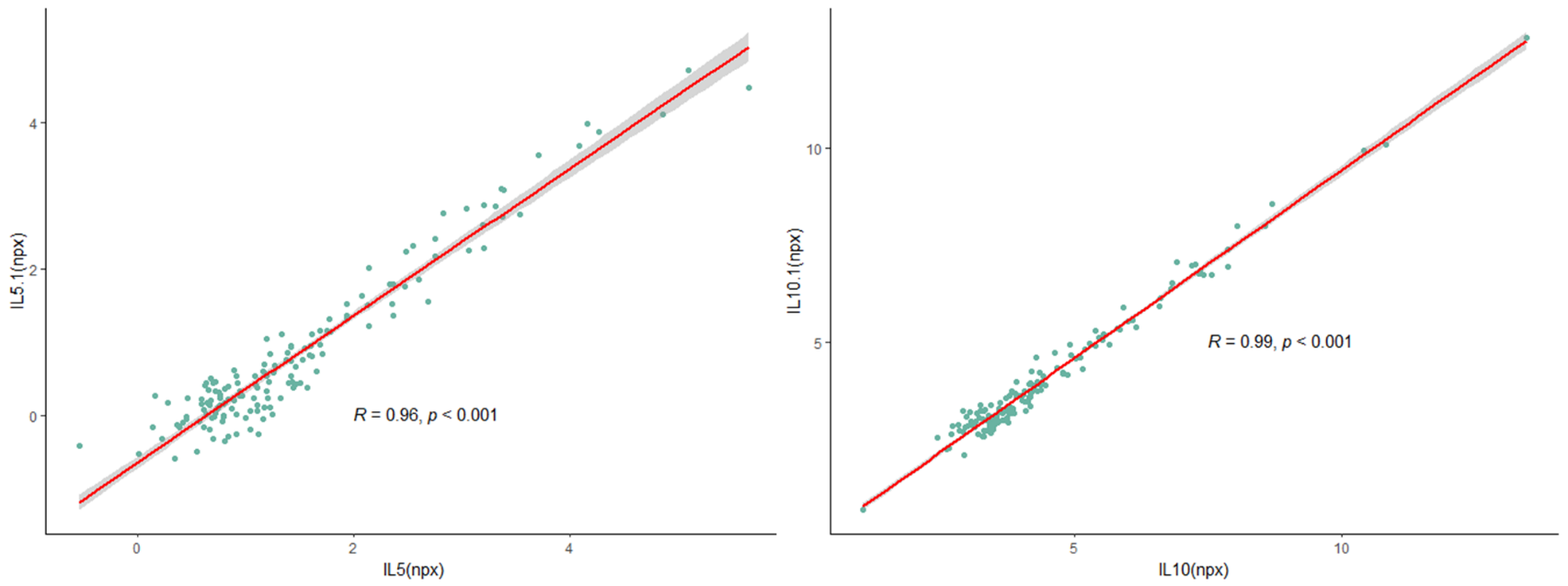

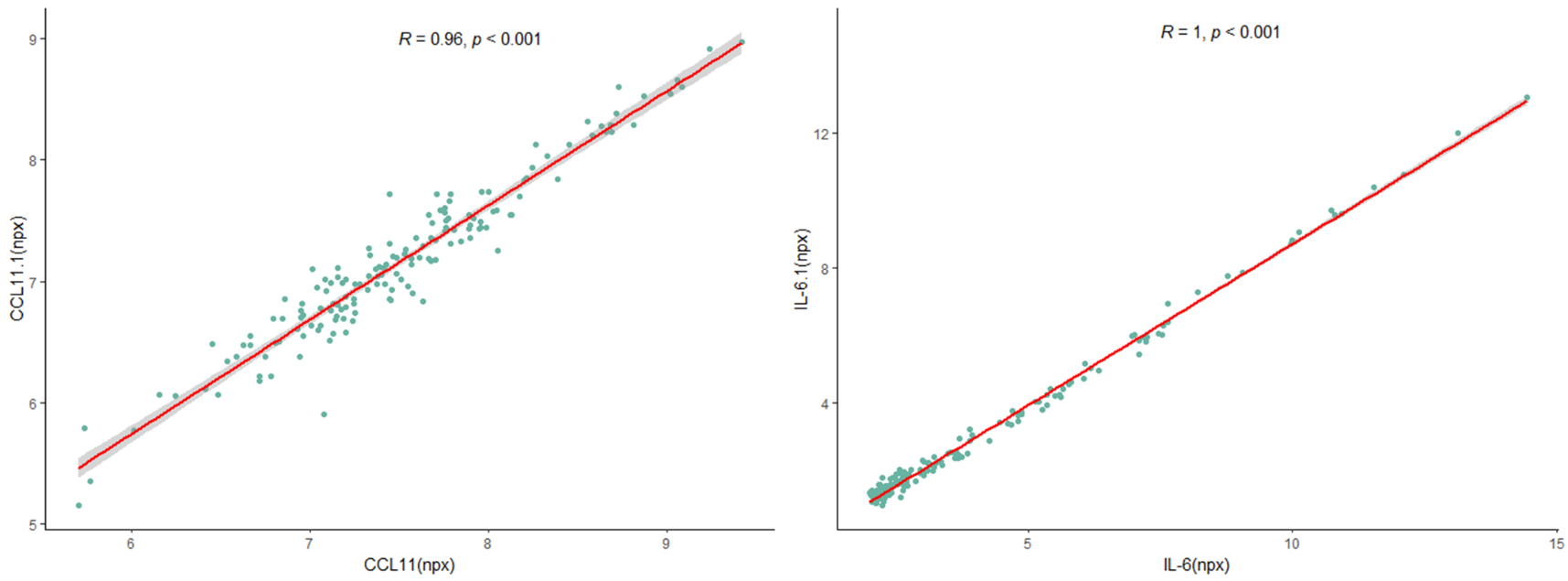
Scatterplot showing Pearson’s correlation of the 4 analytes measured by both PEA panel “Immune response” and PEA panel “Inflammation”. Grey area indicates 95% CI.

Figure 1: Correlation of analytes measured by PEA platforms analysing 92 analytes each


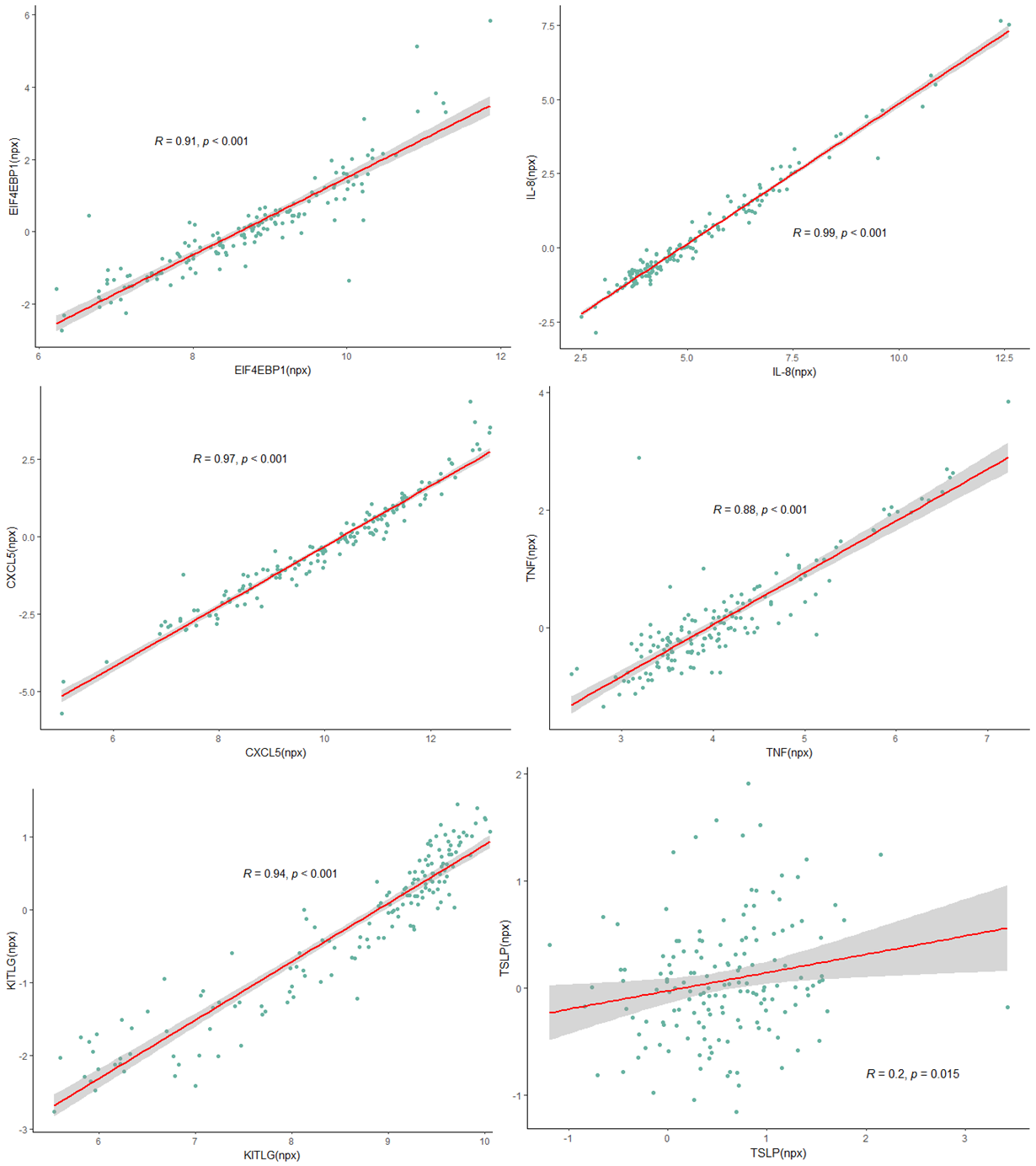


Figure 2: Correlation of analytes measured by PEA platforms

Scatterplots showing Pearson’s correlation of the protein measured by either PEA platform measuring 92 analytes (“Inflammation” or “Immune response”) and a PEA platform measuring 369 analytes (“Cardiometabolic”).

## LC-MS/MS Data

LC-MS/MS analysis led to the quantification of 1,040 proteins in the total cohort of 173 analysed plasma samples. Following data filtering for missing values, 291 proteins were quantified in at least 121 (70%) of the samples (Figure 3).


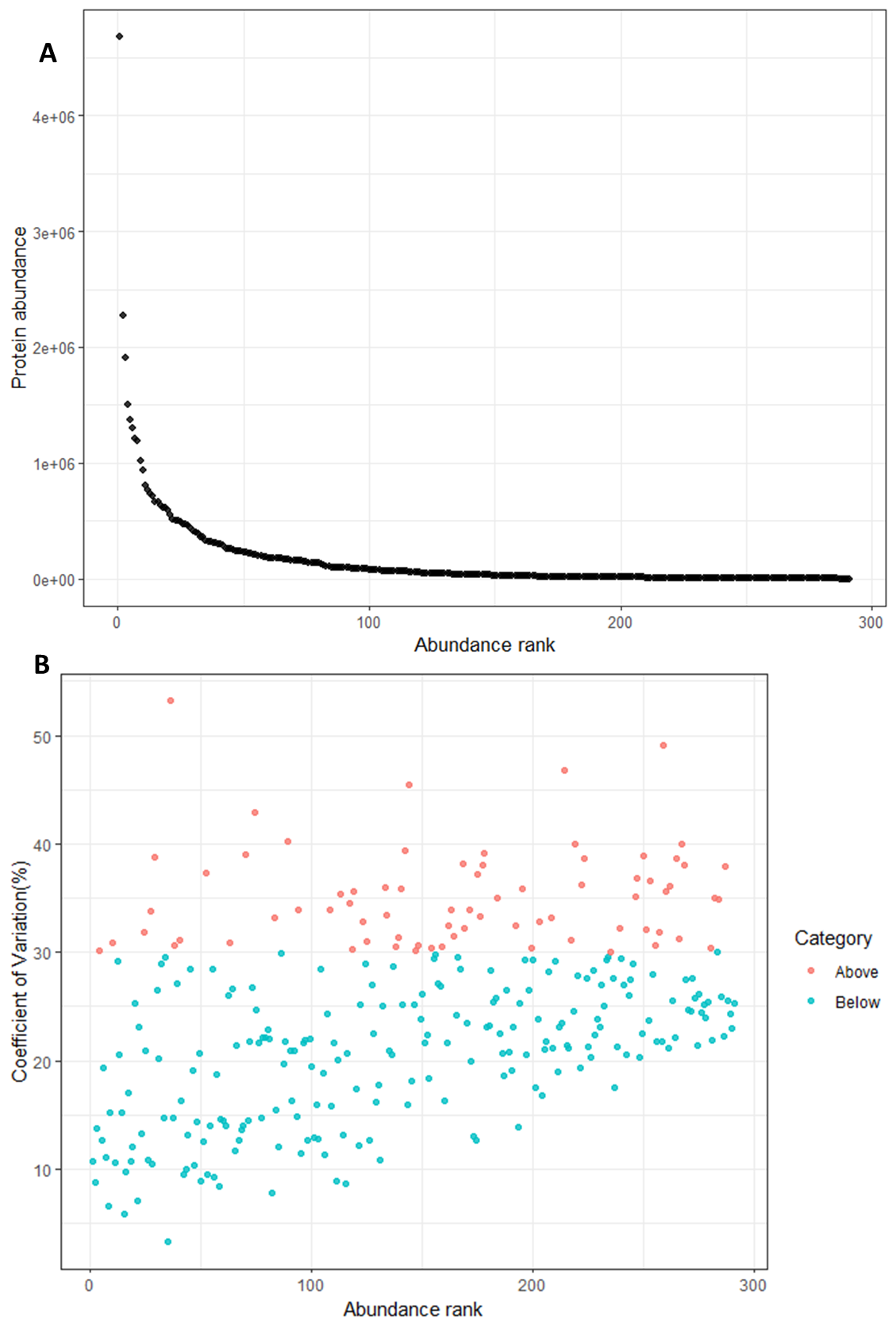


Figure 3: Technical variability of LC-MS/MS analysis

Ranked abundance of 291 proteins quantified by LC-MS/MS. (B) CVs of all quantified proteins were calculated for the nine sample replicate samples, plotted according to abundance. Proteins with CVs < 30% (Below) are coloured in blue and those with CVs >=30% in red (Above).

## LASSO models

Table 1: AUC’s across outer 5 folds of finalised LASSO model using ELISA and ELLA data

| **Fold** | **AUC** |
| --- | --- |
| **1** | 1.00 |
| **2** | 0.80 |
| **3** | 1.00 |
| **4** | 1.00 |
| **5** | 0.92 |

Table 2: Number of times candidate proteins selected in outer 5 fold models

| **Feature** | Number of folds selected in |
| --- | --- |
| **(Intercept)** | 5 |
| **CXCL9** | 5 |
| **PLA2G2A** | 5 |
| **NTproBNP** | 4 |
| **CXCL10** | 1 |

Table 3:LASSO coefficients in finalised model

| **Protein** | **Coefficient** |
| --- | --- |
| CXCL9 | 1.33550806 |
| PLA2G2A | 0.67207431 |
| NTproBNP | 0.80390315 |
| CXCL10 | 0 |

Table 4: Confusion matrix of finalised models performance in ELISA data

|  |  | **Actual** | |
| --- | --- | --- | --- |
|  |  | Febrile | MIS-C |
| **Predicted** | Febrile | 25 | 2 |
|  | MIS-C | 0 | 22 |


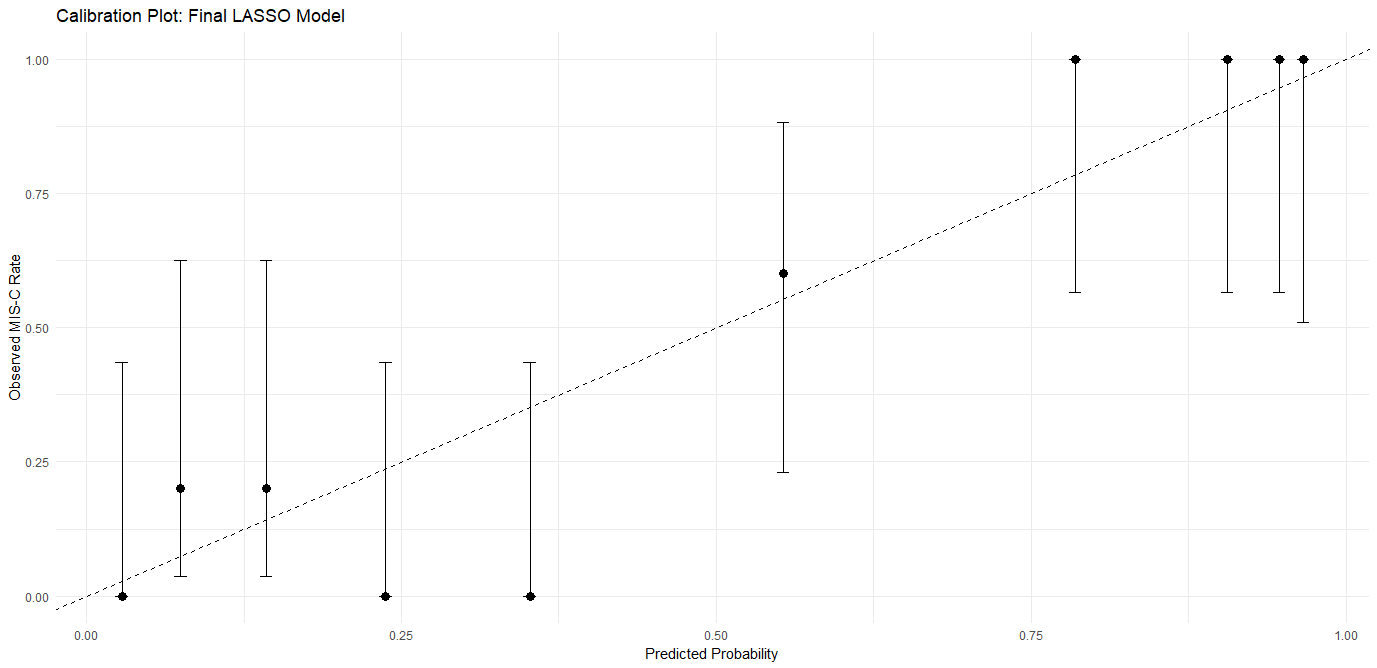


Figure 4: Calibration plot of finalised LASSO model

## LASSO performance in external dataset A

Table 5: Confusion matrix of finalised LASSO model performance in external dataset A

|  | **Actual** | | |
| --- | --- | --- | --- |
|  |  | COVID-19 | MIS-C |
| **Predicted** | COVID-19 | 40 | 3 |
|  | MIS-C | 13 | 31 |
