# Supplementary material for: In depth characterisation of the proteome of MIS-C and post COVID-19 infection in children reveals inflammatory pathway activation and evidence of tissue damage
Source: J Transl Med. 2025 Aug 18;23:929. doi: 10.1186/s12967-025-06826-3 (PMC12363030; doi:10.1186/s12967-025-06826-3)
Supplement: Supplementary file 12 — Supplementary Material 12 [file 12967_2025_6826_MOESM12_ESM.docx]

# Supplementary figures

## Acute COVID-19 Vs Healthy controls


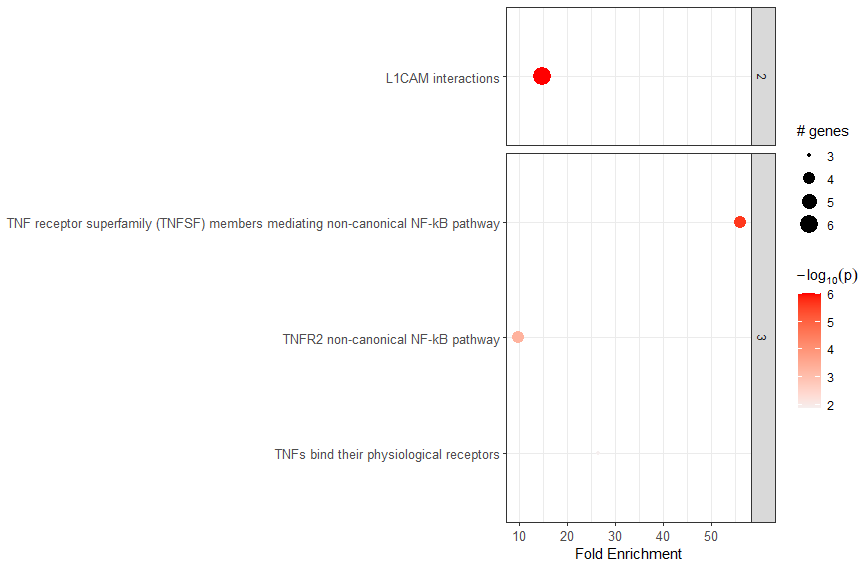


Supplementary figure 1: Enriched pathways in Acute COVID-19 (n=4) using DAP’s identified compared to matched seronegative healthy controls (n=25).

## Seropositive healthy controls vs Seronegative healthy controls


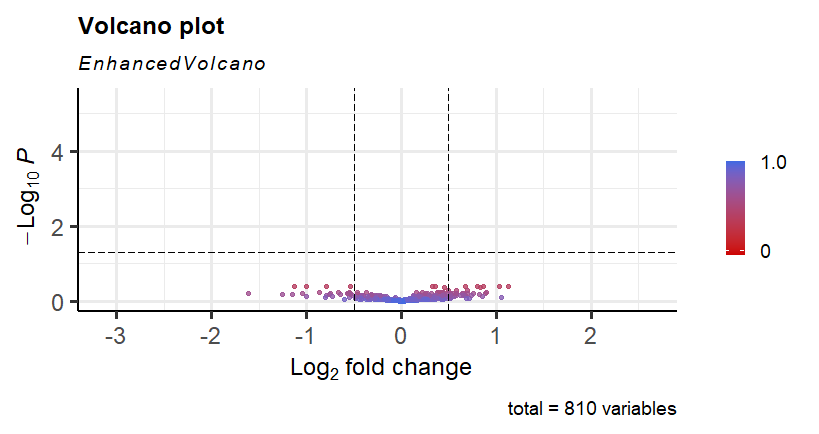


Supplementary figure 2: Volcano plot showing DAPs between seropositive healthy controls (n=25) and seronegative healthy controls(n=25).

## Febrile controls vs matched Healthy controls


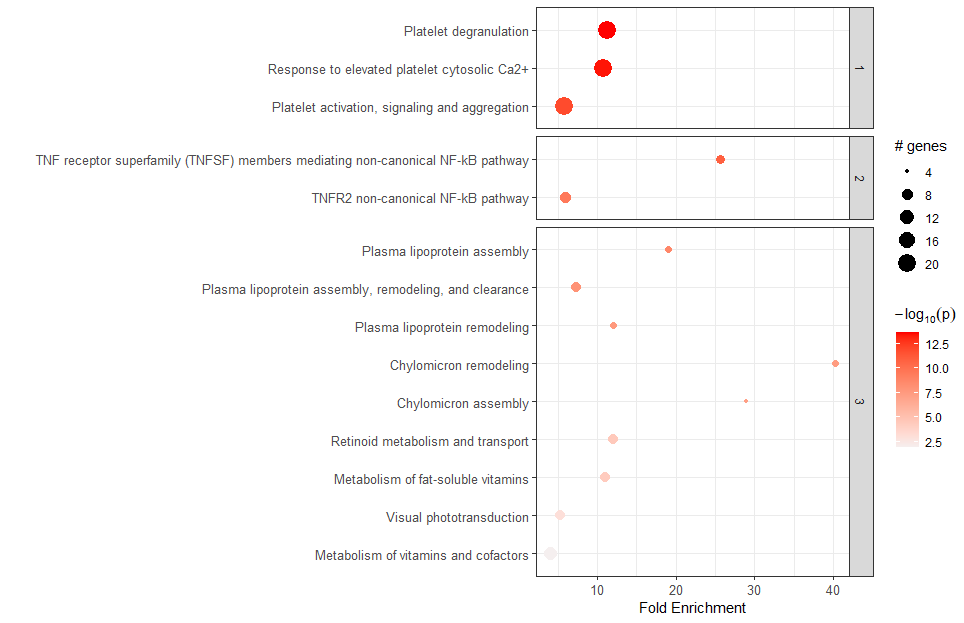


Supplementary figure 3: Enriched pathways in Febrile controls (n=36) using DAP’s identified compared to matched seronegative healthy controls (n=43).

## MIS-C IVIG and ICU


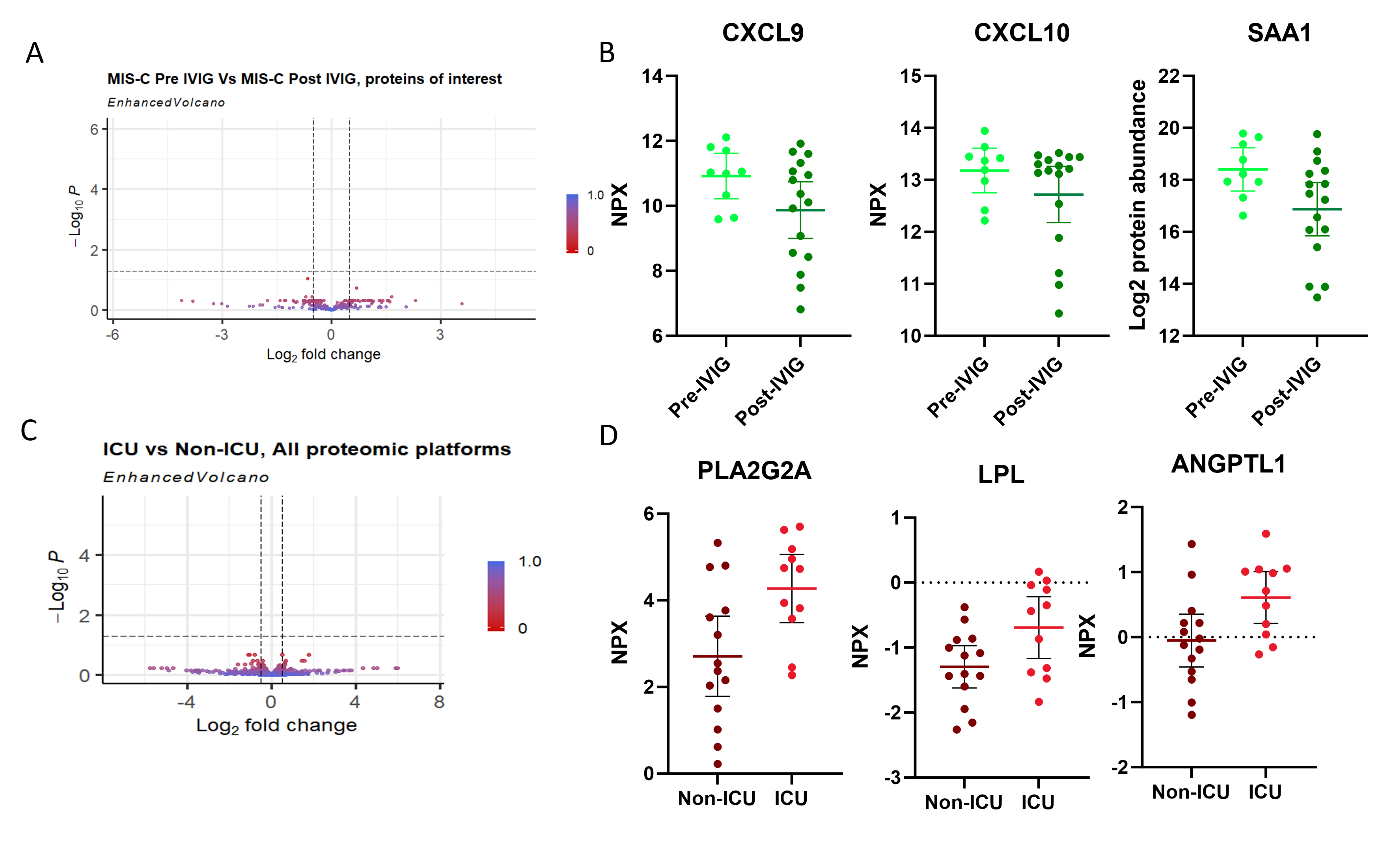


Supplementary figure 4 :Volcano plot showing DAPs between MIS-C cases pre IVIG administration and MIS-C cases post IVIG administration. (B) Dot plots of selected proinflammatory proteins in MIS-C cases pre IVIG administration and MIS-C cases post IVIG administration. (C) Volcano plot showing DAPs between MIS-C cases admitted to ICU and MIS-C cases not admitted to ICU. (D) Dot plots of selected proteins associated with endothelial damage in MIS-C cases pre IVIG administration and MIS-C cases post IVIG administration.

## MIS-C Vs Seropositive Healthy controls


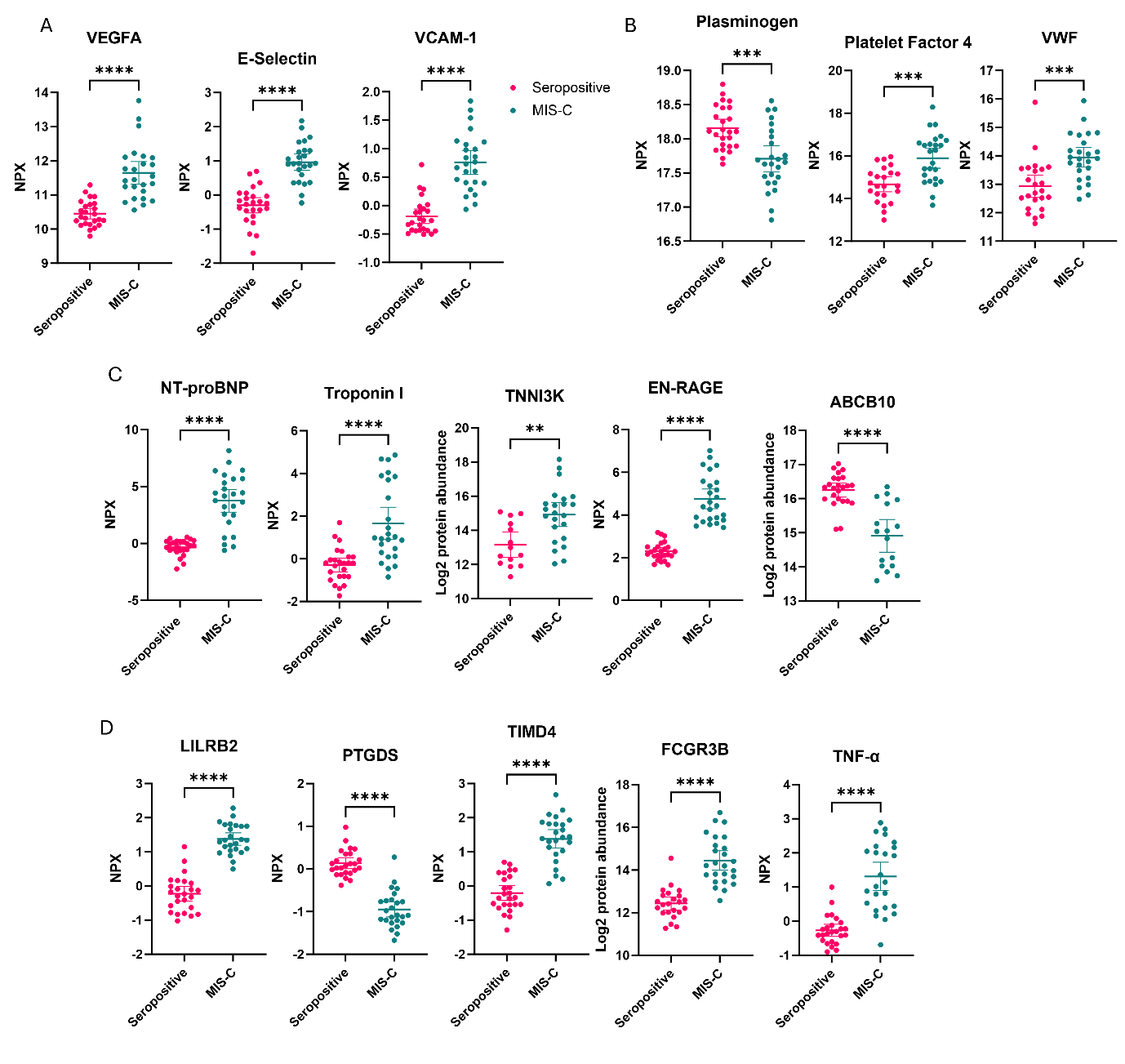


Supplementary figure 5: Selected protein markers associated with (A) endothelial damage, (B) platelet activation, and (C) myocardial injury that are significantly altered in MIS-C compared to seropositive healthy controls. (D) Selected proteins that are significantly altered in MIS-C compared to seropositive healthy controls, but not significantly altered in febrile controls in comparison to matched healthy controls.

## MIS-C Vs Febrile controls


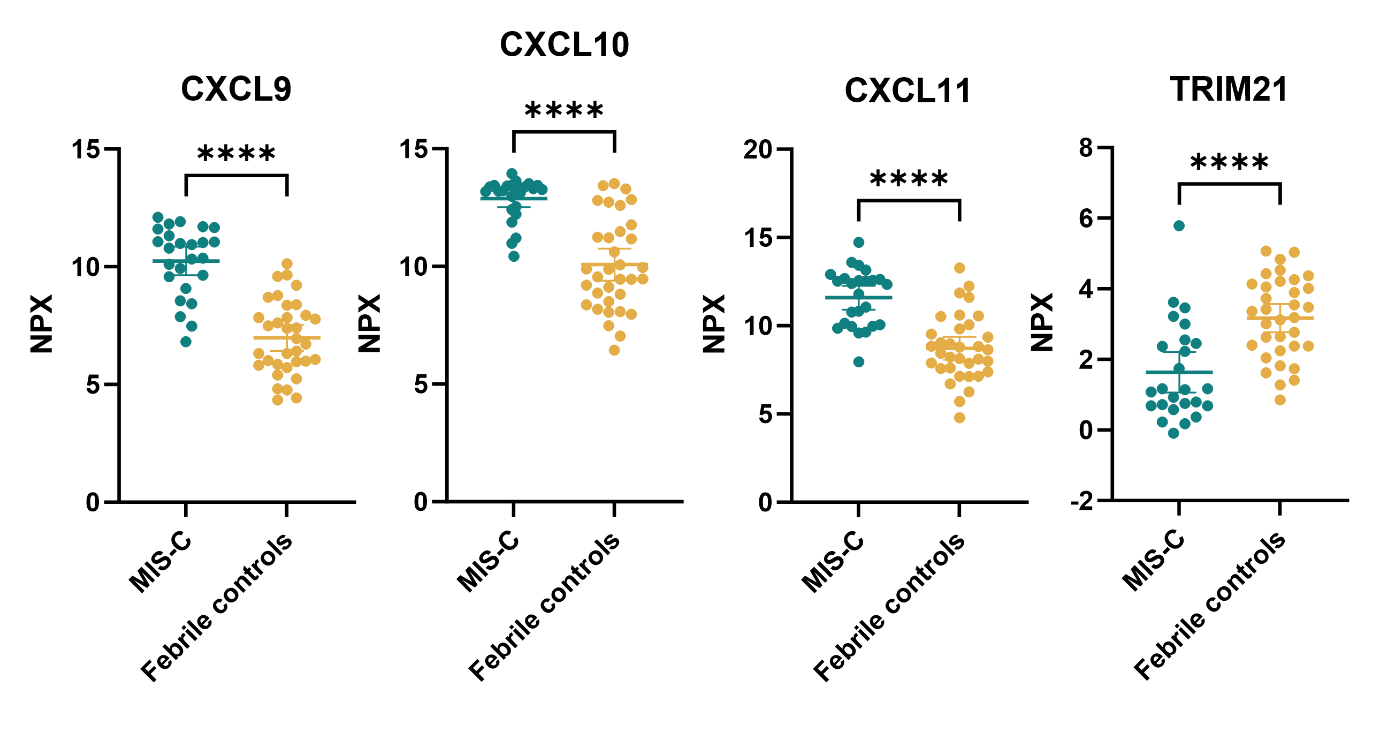


Supplementary figure 6: Pro-inflammatory proteins differentially abundant between MIS-C and febrile controls.

## In silico validation


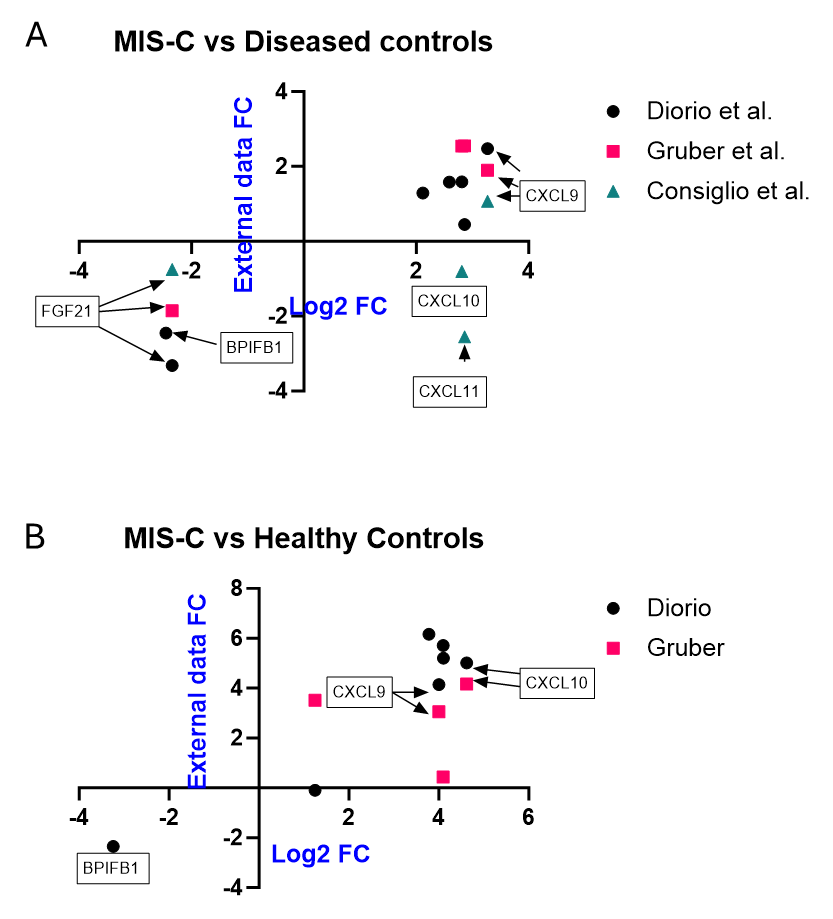


Supplementary figure 7: Correlation of proteins significantly different with a fold change > +/-2 between MIS-C and febrile controls identified in this study with those reported in independent external datasets. Fold changes of top differentially abundant proteins (DAPs) were compared to corresponding values from external studies using similar MIS-C versus diseased control comparisons (B) Correlations of protein fold changes between MIS-C and seropositive healthy controls identified in this study with those reported in independent external datasets. Fold changes of top differentially abundant proteins (DAPs) were compared to corresponding values from external studies using similar MIS-C versus healthy control comparisons.
